# Enhanced self-administration of the CB_1_ receptor agonist WIN55,212-2 in olfactory bulbectomized rats: evaluation of possible serotonergic and dopaminergic underlying mechanisms

**DOI:** 10.3389/fphar.2014.00044

**Published:** 2014-03-20

**Authors:** Petra Amchova, Jana Kucerova, Valentina Giugliano, Zuzana Babinska, Mary T. Zanda, Maria Scherma, Ladislav Dusek, Paola Fadda, Vincenzo Micale, Alexandra Sulcova, Walter Fratta, Liana Fattore

**Affiliations:** ^1^Central European Institute of Technology, Masaryk UniversityBrno, Czech Republic; ^2^Department of Pharmacology, Faculty of Medicine, Masaryk UniversityBrno, Czech Republic; ^3^Section of Neuroscience and Clinical Pharmacology, Department of Biomedical Sciences, University of CagliariMonserrato, Italy; ^4^Institute of Biostatistics and Analyses of Faculty of Medicine, Masaryk UniversityBrno, Czech Republic; ^5^Center of Excellence “Neurobiology of Addiction,” University of CagliariMonserrato, Italy; ^6^National Institute of Neuroscience (INN), University of CagliariMonserrato, Italy; ^7^CNR Institute of Neuroscience-Cagliari, National Research Council-ItalyMonserrato, Italy

**Keywords:** WIN55212-2, cannabinoid, methamphetamine, olfactory bulbectomy, depression, drug dependence, serotonin, dopamine

## Abstract

Depression has been associated with drug consumption, including heavy or problematic cannabis use. According to an animal model of depression and substance use disorder comorbidity, we combined the olfactory bulbectomy (OBX) model of depression with intravenous drug self-administration procedure to verify whether depressive-like rats displayed altered voluntary intake of the CB_1_ receptor agonist WIN55,212-2 (WIN, 12.5 μg/kg/infusion). To this aim, olfactory-bulbectomized (OBX) and sham-operated (SHAM) Lister Hooded rats were allowed to self-administer WIN by lever-pressing under a continuous [fixed ratio 1 (FR-1)] schedule of reinforcement in 2 h daily sessions. Data showed that both OBX and SHAM rats developed stable WIN intake; yet, responses in OBX were constantly higher than in SHAM rats soon after the first week of training. In addition, OBX rats took significantly longer to extinguish the drug-seeking behavior after vehicle substitution. Acute pre-treatment with serotonin 5HT_1B_ receptor agonist, CGS-12066B (2.5–10 mg/kg), did not significantly modify WIN intake in OBX and SHAM Lister Hooded rats. Furthermore, acute pre-treatment with CGS-12066B (10 and 15 mg/kg) did not alter responses in parallel groups of OBX and SHAM Sprague Dawley rats self-administering methamphetamine under higher (FR-2) reinforcement schedule with nose-poking as *operandum*. Finally, dopamine levels in the nucleus accumbens (NAc) of OBX rats did not increase in response to a WIN challenge, as in SHAM rats, indicating a dopaminergic dysfunction in bulbectomized rats. Altogether, our findings suggest that a depressive-like state may alter cannabinoid CB_1_ receptor agonist-induced brain reward function and that a dopaminergic rather than a 5-HT_1B_ mechanism is likely to underlie enhanced WIN self-administration in OBX rats.

## Introduction

Many psychiatric disorders including depression, schizophrenia, and anxiety are frequently associated to drug addiction (Langas et al., 2010; Testa et al., 2013). Recently, clinical associations between depression and marijuana smoking have been reported (Horwood et al., 2012; Lev-Ran et al., 2013). Yet, whether cannabis abuse in depressed patients antedates the disorder onset or is a consequence of its course is still to be determined. Several hypotheses have been offered to explain the high rates of marihuana smoking in people with depression including genetic factors, environmental influences, and self-medication. Recently, a genetically conditioned hypersensitivity to elicit cannabis dependence was evaluated in a depressive population, and although the outcome was not fully conclusive authors suggested that the links between cannabis use and depressive symptoms are conditional on the individual's genetic makeup (Otten and Engels, 2013). Social difficulties such as limited economical resources, impaired interpersonal skills, social isolation, or stressful events may also trigger both depression and cannabis abuse (Baker et al., 2010). On the other hand, cannabis use has been proposed to serve as a self-medication in depressed patients (Degenhardt et al., 2003), although some studies excluded that subjects with prior depression experience symptom relief after smoking cannabis (Arendt et al., 2007).

The self-medication theory was developed on the basis of the monoaminergic hypothesis of depression, according to which depression is associated with a reduced monoaminergic transmission, in particular noradrenaline and serotonin (5-HT) (Rotenberg, 1994; Prins et al., 2011). In fact, symptoms related to monoaminergic deficits (as in depression) may be relieved by a variety of abused drugs (Khantzian, 1985; Hall and Queener, 2007; Becker et al., 2012; Holma et al., 2013). Accordingly, stimulant-dependent patients with depressive disorders reduce their abuse of stimulants when treated with antidepressants to a greater extent than non-depressed (stimulant-dependent) individuals (Markou et al., 1998; Wohl and Ades, 2009). Similar to other abused drugs, cannabis induces release of dopamine (DA) in the mesolimbic reward pathway (Oleson and Cheer, 2012), thus elevating mood and improving wellbeing. However, it also significantly affects bioavailability of serotonin. Notably, genetic deletion of the cannabinoid CB_1_ receptor reduces the functionality of the brain serotonin system (Mato et al., 2007), while chronic CB_1_ receptor antagonism induces a depression-like phenotype (Beyer et al., 2010).

Majority of available studies aimed at investigating the role of endocannabinoid system in animal models of depression reported a decreased activity of the endocannabinoid system (Micale et al., 2013a,b). Pharmacological and genetic blockade of cannabinoid CB_1_ receptors result in symptoms that mimic those seen in depression (Ashton and Moore, 2011). In keeping with this, stimulation of CB_1_ receptors exerts an antidepressant-like effect similar to that induced by the antidepressants desipramine and fluoxetine in the rat forced-swim test (Hill and Gorzalka, 2005) and the olfactory bulbectomy (OBX) rat model of depression (Rodriguez-Gaztelumendi et al., 2009), respectively. These antidepressant effects are antagonized by administration of CB_1_ receptor antagonists leading back to depression-like phenotypes (Hill and Gorzalka, 2005). All these and other preclinical evidence strengthen the involvement of the endocannabinoid system in depressive-like states. However, no data are available on the voluntary consumption of cannabinoid receptor agonists in animal models of depression.

The multi-faceted effects of 5-HT are mediated by at least 14 receptor subtypes (Hoyer et al., 1994). Among them, the 5-HT_1B_ receptor subtype has recently attracted scientific attention for its potential role in modulating addictive behaviors (Pentkowski et al., 2012; Neisewander et al., 2013). Serotonin 5-HT_1B_ receptors are widely distributed in the brain where function as both autoreceptors and heteroreceptors, that mediate release of serotonin and other neurotransmitters (Barnes and Sharp, 1999; Moret and Briley, 2000; Pytliak et al., 2011; Cai et al., 2013). A number of human and animal studies demonstrated a causal link between altered 5-HT_1B_ receptor activity and development of neuropsychiatric conditions, including depression and drug addiction. For example, lower functioning of 5-HT_1B_ receptors was found in patients suffering major depressive disorders (Murrough et al., 2011), while a polymorphism at the 5-HT_1B_ receptor gene (*HTR1B*) was reported to be associated significantly with alcoholism (Lappalainen et al., 1998). In animal models of drug addiction, stimulation of the 5-HT_1B_ receptor was shown to induce antidepressant effects (Tatarczynska et al., 2004) and to decrease the number of behavioral responses for alcohol (Grant et al., 1997; Maurel et al., 1999; Tomkins and O'Neill, 2000), d-amphetamine (Fletcher and Korth, 1999), intracranial self-stimulation (Hayes et al., 2009) and the positive reinforcing effects of cocaine in rats (Harrison et al., 1999). More importantly, administration of the 5-HT_1B_ receptor agonist CGS-12066B into the nucleus accumbens (NAc) core was shown to decrease operant responses for ethanol but not sucrose solution in rats (Czachowski, 2005) indicating a selective effect of this compound on drug-induced responses. However, systemic administration of CGS-12066B did not reduce cocaine self-administration in rats (Parsons et al., 1996), which implies a certain drug selectivity of this compound in attenuating self-administration behavior.

The main aim of this study was to investigate the intravenous self-administration of the CB_1_ receptor agonist WIN55-212,2, (WIN), using lever-pressing as *operandum* under a continuous [fixed ratio 1 (FR-1)] schedule of reinforcement, in a well-established rat model of depression, the bilateral OBX. Given the significant differences observed in WIN self-administration between OBX and SHAM rats, we decided to perform pilot experiments in an attempt to shed some light on possible underlying mechanisms. Therefore, since the 5-HT_1B_ receptor has been recently involved in the modulation of both depression and drug intake, we tested the effect of the 5-HT_1B_ agonist CGS-12066B (CGS) on WIN self-administration in OBX and sham-operated (SHAM) Lister Hooded rats displaying depressive-like phenotypes. To further investigate the role of 5-HT_1B_ receptor in drug-taking behavior and verify its effect on the self-administration of a different drug, in different strains of rats and under dissimilar experimental conditions, we tested the CGS compound in OBX and SHAM Sprague Dawley rats self-administering methamphetamine (METH) as previously reported (Kucerova et al., 2012). CGS was chosen because of its high selectivity to 5-HT_1B_ receptors (Neale et al., 1987) and its reducing effects on amphetamine (Fletcher and Korth, 1999) and alcohol self-administration (Tomkins and O'Neill, 2000; Czachowski, 2005). Finally, since cannabinoid self-administration was shown to be associated to an increased DA transmission in the shell of the NAc (Fadda et al., 2006), we used the *in vivo* microdialysis technique to test whether OBX and SHAM rats displayed similar increase in DA levels within the NAc shell in response to a challenge of WIN at a dose (0.3 mg/kg) mimicking daily mean amount of the drug typically self-administer by trained rats.

## Materials and methods

### Animals

Adult male Lister Hooded rats weighting 250–270 g at the beginning of the experiment (9 weeks old) were purchased from Harlan-Nossan (Italy) and housed four per cage at the Animal Facility of the Department of Biomedical Sciences, University of Cagliari, Italy. Rats were provided with free access to water and food and maintained on a reversed 12/12 h light/dark cycle (lights on 7 p.m.) with constant room temperature (22 ± 2°C) and humidity (60%). The experimental protocols were approved by the local Animal Care Committee at the Department of Biomedical Sciences, University of Cagliari, Italy.

Adult male albino Sprague Dawley rats weighting 220–240 g at the beginning of the experiment (8 weeks old) were purchased from Charles River (Germany) and housed individually at the Animal Facility of the Department of Pharmacology, Masaryk University in Brno, Czech Republic. Animals were maintained on a reversed 12/12 h light/dark cycle (lights on 5 p.m.) with constant relative humidity of 50–60% and temperature of 23 ± 1°C, and food and water available *ad-libitum*. The experimental protocols were approved by the Animal Care Committee of the Faculty of Medicine, Masaryk University, Czech Republic.

All experiments were carried out in strict accordance with the E.C. Regulations for Animal Use in Research (CEE No. 86/609) and local acts.

### Drugs and treatments

For self-administration training, WIN55-212,2 (R-[2,3-dihydro-5-methyl-3-[(morpholinyl) methyl]-pyrrolo[1,2,3-de]-1,4-benzoxazinyl]-(1-naphthalenyl)-methanone mesylate), (WIN, RBI, USA) was freshly dissolved in one drop of Tween 80, diluted in heparinized (1%) saline solution and made available at the dose of 12.5 μg/kg/infusion (volume of infusion: 100 μl), as previously described (Fattore et al., 2001). To ensure sterility, fresh drug solutions were filtered by 0.22 μm syringe filters prior to use. For microdialysis testing, WIN solution was prepared as described above and administered intravenously at the dose of 0.3 mg/kg (volume of injection: 1 ml/kg). This drug dose was selected on the basis of the daily amount of WIN typically self-administered by male Lister Hooded rats under the same experimental conditions (Deiana et al., 2007; Fattore et al., 2007; Spano et al., 2010). Importantly, this dose of WIN was also shown to significantly increase DA release in the shell part of the NAc of rats (Tanda et al., 1997).

Methamphetamine (METH, Sigma Chemical Co., St Louis, MO, USA) was dissolved in saline sterile solution and made available at dose of 0.08 mg/infusion as previously described (Vinklerova et al., 2002).

CGS-12066B, 7-trifluoromethyl-4(4-methyl-1-piperazinyl)-pyrrolo[1,2-a]-quinoxaline dimaleate (CGS, R&D systems, Abingdon, Oxon, UK) was dissolved in saline and administered intraperitoneally (i.p.) at doses ranging from 2.5 to 15 mg/kg (volume of injection: 2 ml/kg), and administered 20 min before starting the session. These drug doses were selected on the basis of their ability to acutely reduce self-administration behavior in rats in a dose-dependent manner (Parsons et al., 1996). Treatments were assigned on the basis of a Latin square design whereby at least three training sessions separated two consecutive testing sessions to allow for assessment of carryover effects. Each animal was tested once with each drug dose and once with saline in a counterbalanced manner, i.e., the order of presentation of different treatments was varied between animals.

All antibiotics and anesthetics were purchased as sterile solutions from local distributors.

### Olfactory bulbectomy (OBX) surgery

At the beginning of the behavioral and neurochemical experiments, rats were randomly divided into two groups: OBX and SHAM rats. The bilateral ablation of the olfactory bulbs was performed as previously described (Kucerova et al., 2012). Animals were anaesthetized with isofluran 2% (Italy) or i.p. injections of 50 mg/kg ketamine plus 8 mg/kg xylazine (Czech Republic). The top of the skull was shaved and swabbed with an antiseptic solution. Then, midline frontal incision was made on the skull and the skin was retracted bilaterally. Two burr holes, 2 mm in diameter, were drilled in the frontal bone 7 and 7.5 mm anterior from the bregma, 1.5 and 2 mm lateral to bregma suture for rats weighing 230 ± 10 g and 260 ± 10 g, respectively. Both olfactory bulbs were removed by aspiration paying particular attention to not damage the frontal cortex. Prevention of blood loss from the ablation cavity was achieved by filling the dead space with a hemostatic sponge. The skin above the lesion was closed with suture. Finally, bacitracin plus neomycin powder was applied to prevent bacterial infection. SHAM rats underwent identical anesthetic and drilling procedures but their bulbs were left intact.

A period of at least 20 days was allowed for the recovery from the surgical procedure and the development of the depressive-like syndrome. During this period, animals were handled daily for few minutes to eliminate aggressiveness, which could otherwise arise (Leonard and Tuite, 1981; Song and Leonard, 2005). Before starting either drug self-administration training or microdialysis experiments, animals were tested in the sucrose preference and motor activity test for anhedonia and hyperactive locomotor response to a novel environment, respectively, (Song and Leonard, 2005).

### Sucrose preference test

After 20 days of recovery from the OBX surgery, Lister-Hooded animals were transferred into single housing with free access to food. A two-bottle choice procedure was used to determine baseline sucrose intake. During the 24-h training phase, all rats were provided in their home cage with two water bottles on the extreme sides of the cage to adapt for drinking from two bottles. After training, one bottle was randomly switched to contain 2% sucrose solution, a concentration known to provide a robust sucrose preference (Muscat and Willner, 1989). The side of sucrose presentation in the home cage was counterbalanced across rats. At 4 and 24 h time intervals both bottles were removed and the amount of liquid remaining in each bottle was measured. After 4 h, the relative position of the bottles was inverted, i.e., they were switched from one side of the cage to the other to avoid perseveration effects. The sucrose preference score was calculated as the percentage of sucrose solution ingested relative to the total amount of liquid consumed as determined before and after each test, i.e., sucrose preference = sucrose intake/total liquid (sucrose + water) intake × 100.

### Locomotor activity test

A day after conclusion of the sucrose preference test, the validity of OBX lesions was further confirmed by assessing increased activity in a brightly lit novel environment. Rats were individually tested for locomotor activity using the Digiscan Animal Activity Analyser (Omnitech Electronics, USA) as previously described (Castelli et al., 2013). Each operant cage (42 × 30 × 60 cm) was equipped with two sets of 16 photocells located at right angles to each other projecting horizontal infrared beams 2.5 cm apart and 2 cm above the cage floor. The outside of the four walls was covered with aluminium foil and two 90-W light bulbs were located at diagonally opposed corners to provide bright illumination. Rats were brought into the testing room individually, placed in the center of the box, and allowed to move freely for 10 min. Locomotor activity was defined from measurement of sequential infrared beam breaks recorded at every 5 min after placing the animals individually in the cage. During the 10-min test the following behavioral parameters were measured:

- Horizontal activity: The total number of beam interruptions that occurred in the horizontal sensors;
- Vertical activity: The total number of beam interruptions that occurred in the vertical sensors; that is the number of times the animal rose onto its hind legs with the front limbs either against the wall or freely in the air (number of rearing episodes);
- Total distance (cm): The horizontal distance travelled by the animal (dependent on animal's trajectory).

At the end of the session, animals were gently removed from the Plexiglas boxes and returned to their home cage. Boxes were wiped with H_2_O_2_ between sessions to prevent olfactory cues.

### Intravenous drug self-administration surgery

At the end of the motility test, OBX and SHAM animals were deeply anesthetized with isofluran 2% (in Italy) or i.p. injections of 50 mg/kg ketamine plus 8 mg/kg xylazine (in the Czech Republic). Under aseptic conditions, a permanent intracardiac silastic catheter was implanted through the external jugular vein to the right atrium. The outer part of the catheter exited the skin in the midscapular area. After surgery, each animal was allowed for recovery, individually, in its home cage with food and water freely available. On the following 6–7 days, each rat received an intravenous infusion of gentamicin (0.16 mg/kg, Italy) or heparinized cephazoline (Vulmizolin 1.0 g, Czech Republic) solution followed by 0.1 ml of a heparinized (1%) sterile saline solution to prevent infection and occlusion of the catheter. During recovery, changes in general behavior and body weight were monitored. When a catheter was found to be blocked or damaged, the animal was excluded from the analysis. Once completely recovered from surgery, food-restriction condition was applied, where rats were fed with 20 g/day of standard rat chow given in the home cage immediately after each session.

### Intravenous self-administration

WIN55-212,2 self-administration was conducted in 12 operant chambers (29.5 × 32.5 × 23.5 cm, Med Associates, Vermont, USA) using lever-pressing as *operandum* under a continuous (FR-1) schedule of reinforcement, i.e., each active response was reinforced. Each chamber was encased in a sound and light attenuating cube. In addition, chambers had a ventilation fan, and a front panel equipped with two retractable levers (each 4 cm wide) positioned 12 cm apart, 8 cm from the grid and extending 1.5 cm into the box. A white stimulus light was placed above each lever and a red house light was located on the opposite wall. Intravenous infusions of WIN were delivered by a software-operated infusion pump (Med Associates, Vermont, USA) through a counterbalanced single-channel swivel and an extra length of plastic tubing enclosed in a metal spring connecting the swivel to the catheter fitting on the animal's back. Pressure on the lever, defined as active, resulted in: (i) extinction of the house light and illumination of the stimulus light above the active lever for 15 s; (ii) retraction of both levers; and (iii) activation of the infusion pump for 5.8 s delivering 0.1 ml intravenous infusion of drug solution. On completion of the 15 s timeout period, levers were re-extended into the chamber, stimulus light went out and the house light was switched on. Pressure on the other lever, defined as inactive, was not coupled to any successive event, but was always recorded to provide an index of basal activity levels. Assessment of schedules and data collections was programmed by means of a computer using the MED Associates MED-PC software package. Throughout each phase of the study, locomotor activity was monitored within the operant chambers, which were equipped with a series of photocells located 3.5 cm above the cage floor. The number of photocell beam breaks was recorded and served as a measure of general horizontal locomotor activity. Self-administration sessions lasted 120 min and took place 7 days/week between 9 a.m. and 1 p.m. during the dark period of the cycle.

Acquisition training was carried out until steady baseline of drug intake was reached. Response was considered stable when animals displayed accurate discrimination between the active and inactive lever. Acquisition was defined as the number of active lever-presses >15 and not differing by more than 20% for three consecutive days. Rats not meeting the acquisition criterion were excluded from the subsequent phases of the study. Only rats developing a stable pattern of WIN intake were allowed to continue daily self-administration sessions until day 30. Then, extinction condition was introduced by replacing WIN with sterile vehicle solution (1% Tween 80 in saline solution) which allowed responses to be recorded without drug consequences. All other experimental parameters were left unchanged; therefore pressure on the active lever resulted in an infusion of 0.1 ml of vehicle accompanied by the presentation of the stimulus light previously paired with WIN delivery. Drug-reinforced behavior was considered extinguished when the maximum number of responses on the active lever was ≤10 and the total number of lever presses (i.e., active + inactive) in a single test session was ≤20.

Methamphetamine self-administration was conducted as previously described (Kucerova et al., 2009, 2012) in 10 standard experimental (30 × 25 × 30 cm, Coulbourn Instruments, USA) boxes using nose-poking as *operandum* under a final FR-2 schedule of reinforcement, i.e., animal had to make two consecutive nose-pokes on the active hole to obtain a single drug infusion. Each cage was provided with two nose-poke holes allocated on one side and programmed by software Graphic State Notation 3.03 (Coulbourn Instruments, USA). Specifically, training sessions were initially conducted under a FR-1 schedule of reinforcement. Fixed-ratio requirement was then raised to FR-2 when the animal fulfilled the following conditions for three consecutive sessions: (a) at least 70% preference of the drug-active nose-poke, (b) minimum intake of 10 infusions per session, or (c) stable intake of the drug (maximum 10% deviation). Nose-pokes in the active hole led to the activation of the infusion pump and administration of a single infusion followed by a 10 s timeout, while nose-pokes in the inactive hole were recorded but not rewarded and reset the count of active nose pokes back to zero. The cage was illuminated by a house light during the session. The light was flashing when administering infusion and off during the time-out period. Self-administration sessions lasted 90 min and took place 7 days/week between 7 a.m. and 2 p.m. during the dark period of the cycle. Acquisition criteria were the same as for WIN self-administration behavior.

In both apparatuses, assignment of the active (drug-paired) and the inactive (not drug-paired) levers/holes was counterbalanced between rats and remained constant for each subject throughout all the experiments. CGS testing was performed on Lister Hooded and Sprague Dawley rats self-administering WIN and METH, respectively, during the maintenance phase of the self-administration training, i.e., once animals stabilized their drug intake.

### Microdialysis surgery and procedure

A separate batch of drug-naïve Lister Hooded male rats was used for the *in vivo* microdialysis study. Rats were anesthetized with 2% isoflurane and placed in a stereotaxic frame (David Kopf Instruments, Tujunga, CA, USA). The skull was exposed and a small hole was drilled on the right side. A concentric self-made microdialysis probe with a 2 mm dialyzing surface length (AN 69AF; Hospal-Dasco, Bologna, Italy; cut-off 40,000 Da, *in vitro* recovery about 30%) was inserted vertically into the shell of the NAc (coordinates from bregma, AP: +1.7, L: ±0.7, V: −8.2) according to the Paxinos anatomical atlas (Paxinos and Watson, 1998) and then fixed to the skull using dental acrylic cement. During the same surgery session, rats were implanted with intravenous catheters as previously described, which allowed intravenous administration of WIN. Starting 24 h from implantation of the dialysis probe, artificial cerebrospinal fluid (147 mM NaCl, 4 mM KCl, 1.5 mM CaCl2, pH 6–6.5) was pumped through the dialysis membrane at a constant rate of 2.5 μ L/min with a CMA/100 microinjection pump (Carnegie Medicine, Sweden). Dialysate samples (50 μ L) were collected every 20 min and directly injected into a high performance liquid chromatography system in order to quantify DA. The system consisted of an isocratic pump (ESA model 580; ESA, Chelmsford, Massachusetts), a 7125 Rheodyne injector connected to a Hewlett Packard (Waldbronn, Germany) series 1100 column thermostat with a reverse phase column (LC18 DBSupelco, 5 μm, 4.6 × 150 mm), and an ESA Coulochem II detector. The first electrode of the detector analytical cell was set at 400 mV and the second at −180 mV; column temperature was set at 30°C. The mobile phase, delivered at 1.0 mL/min, consisted of 50 mM/L sodium acetate, 0.073 mM/L Na2 ethylenediaminetetraacetic acid, 0.35 mM/L 1-octanesulfonic acid, 12% methanol, pH 4.21, with acetic acid. In this condition the sensitivity of the assay for DA was 2 fM/sample. Only results deriving from rats with correctly positioned dialysis probes were included in statistical analysis of data. The location of the probe was determined histologically at the end of each experiment by examining coronal brain sections (50 μm) stained with cresyl violet.

### Statistical data analysis

At the end of the study, rats were anesthetized by isofluran inhalation and decapitated. Their brains were removed for confirmation of the ablation of the olfactory bulbs. Only rats with a complete removal of both olfactory bulbs with no damages to the frontal cortex were included for data analysis.

Primary data were summarized using arithmetic mean and standard error of the mean estimate. Statistical analysis of differences between OBX and SHAM rats in time-related differences used One-Way and Two-Way analysis of variance (ANOVA) with repeated measures model. In Two-Way repeated measures ANOVA, the group of rats was entered as the between-group factor and the time-points as repeated within-subject measure. One-Way ANOVA was used when comparing the time-points within given experimental group of rats.

Time-related changes and differences between OBX and SHAM groups during drug self-administration after acute pre-treatment with the 5-HT1B receptor agonist CGS-12066B were analyzed using Two-Way repeated measures ANOVA.

Microdialysis data were analyzed using One-Way or Two-Way ANOVA (treatment × time), followed by Tukey's or Bonferroni's *post-hoc* test comparison procedures, respectively. The level of statistical significance was set at *p* < 0.05.

Statistics were calculated using the statistical package SPSS (version 2.0).

## Results

### Behavioral profile after OBX surgery (lister hooded rats)

To verify the development of a depressive-like phenotype in OBX lesioned animals, basal behavioral differences between SHAM and OBX rats were established by measuring sucrose preference and locomotor activity. Anhedonia and hyperactive response to a novel brightly lit environment are two major features of OBX rats consistently described by previous studies (Kelly et al., 1997; Song and Leonard, 2005; Romeas et al., 2009).

**Figure 1:**
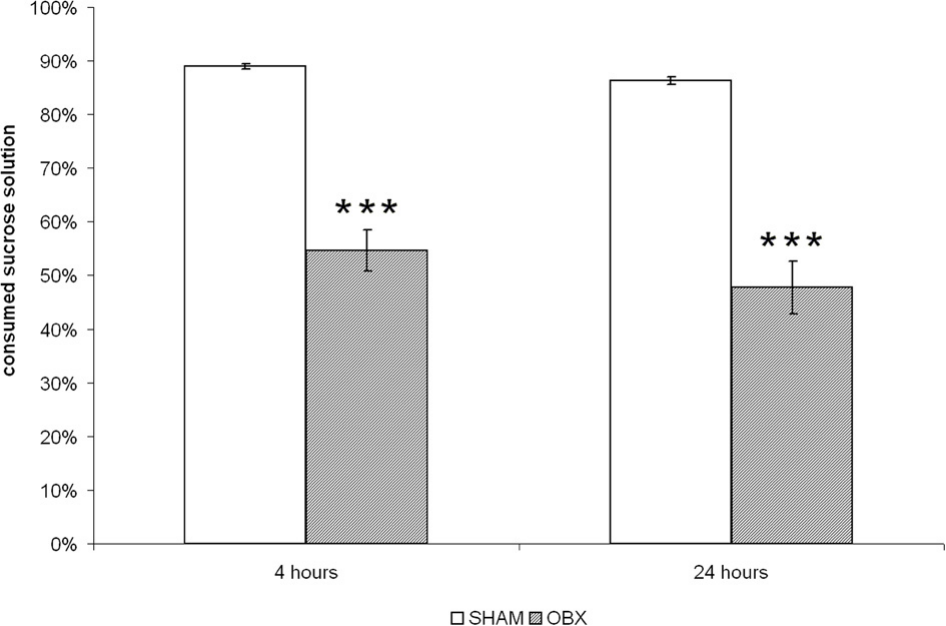
**OBX rats display anhedonia**. The sucrose preference test in SHAM (*n* = 6) and OBX (*n* = 7) Lister Hooded rats. Data are percentages (±s.e.m.) of sucrose solution consumed at two time-points, i.e., after 4 and 24 h from the beginning of the test. Student *t*-test, ^***^p < 0.001.

As shown in Figure 1, OBX rats consumed significantly lower proportion of sucrose than SHAM rats (*p* < 0.001) after both 4 and 24 h from sucrose solution presentation, which confirmed a reduced hedonic response as a consequence of ablation of the olfactory bulbs. Differences between the two dependent variables were tested using independent Student *t*-test.

**Figure 2:**
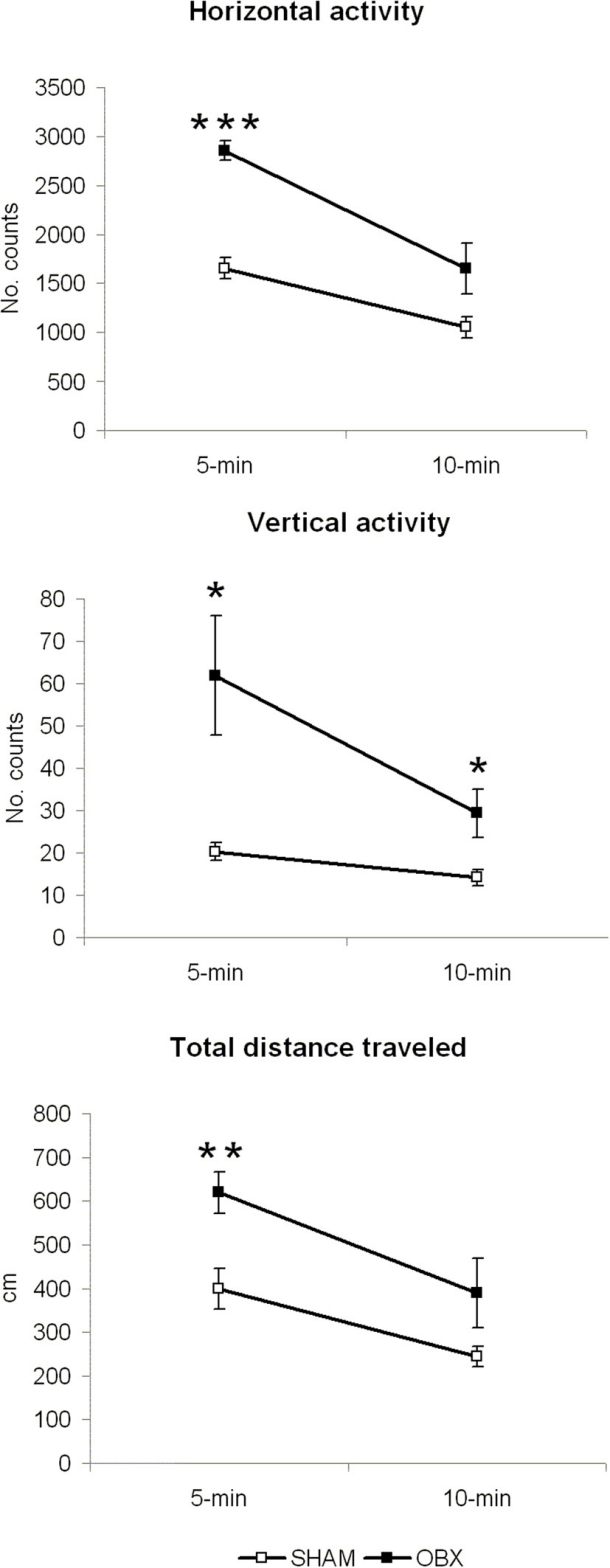
**OBX rats display hyperactivity in a novel aversive environment**. Spontaneous horizontal and vertical activity, expressed as mean counts of photobeam interruptions, and total distance travelled (in centimeters) in SHAM (*n* = 6) and OBX (*n* = 7) Lister Hooded rats. Data are shown as means (±s.e.m.) at two time-points (5 and 10 min of measurement). Student *t*-test, ^*^p < 0.05, ^**^p < 0.01, and ^***^p < 0.001.

Figure 2 illustrates results from the motor activity test conducted in brightly lit conditions. In all locomotor measures (i.e., horizontal activity, vertical activity, and total distance travelled), statistically significant differences were detected at 5 min of measurement, a time-point representing response to novel environment (horizontal activity, *p* < 0.001; vertical activity, *p* < 0.05; total distance travelled, *p* < 0.01). At the 10 min time-point (little effect of novelty), a significant difference was only present in vertical activity measure (*p* = 0.032). Differences between the two independent variables were tested using independent Student *t*-test.

### WIN 55-212,2 self-administration in lister hooded rats

**Figure 3:**
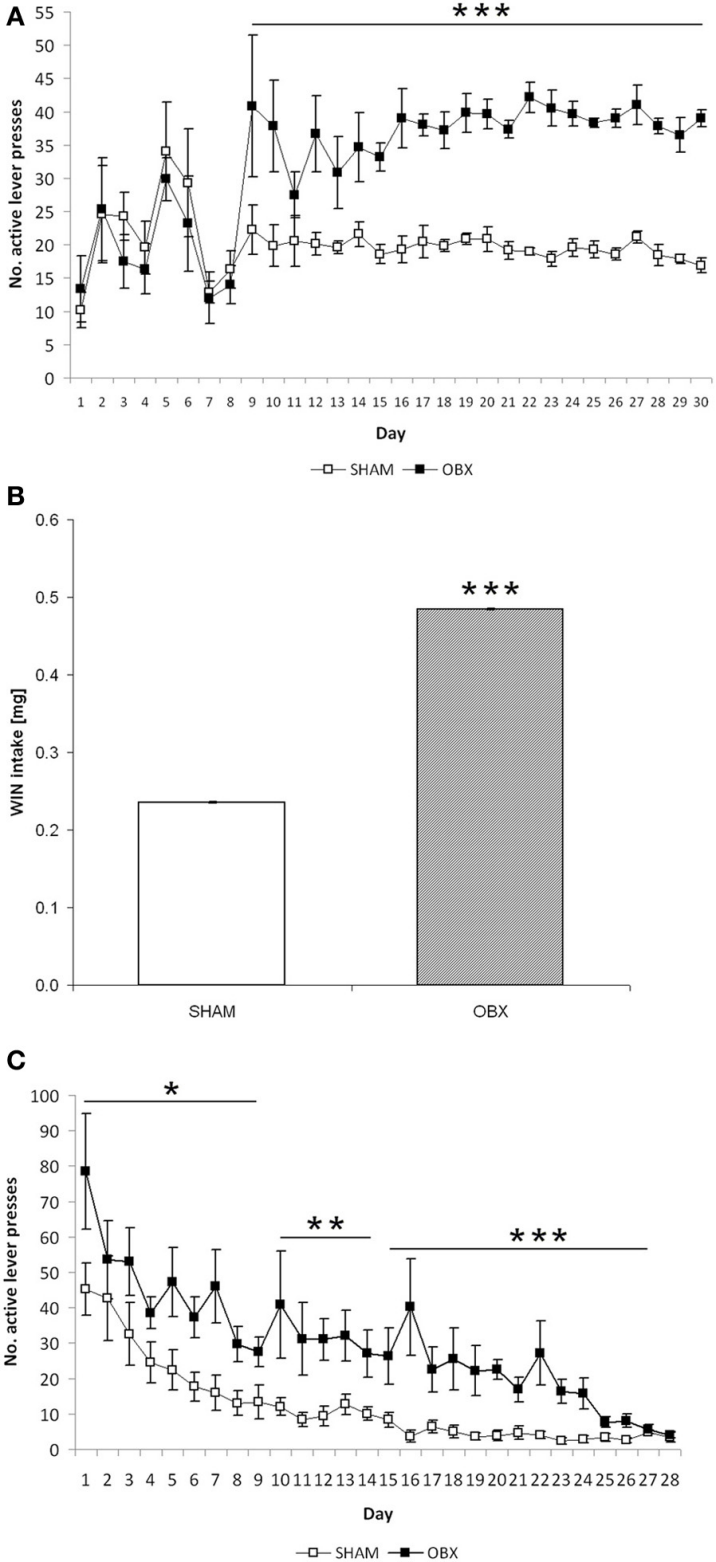
**Cannabinoid self-administration training in OBX and SHAM rats**. Data are shown as means (±s.e.m.). **(A)** Acquisition phase: mean active lever presses in SHAM (*n* = 6) and OBX (*n* = 7) Lister Hooded rats during WIN55,212-2 self-administration. ^***^α < 0.001, repeated measures ANOVA. **(B)** Maintenance phase: mean WIN intake over the last seven training sessions in SHAM (*n* = 6) and OBX (*n* = 7) Lister Hooded rats during WIN self-administration. ^***^α < 0.001, repeated measures ANOVA. **(C)** Extinction phase: mean active lever presses in SHAM (*n* = 6) and OBX (*n* = 7) Lister Hooded rats during extinction of WIN self-administration. ^*^α < 0.05, ^**^α < 0.01, and ^***^α < 0.001, repeated measures ANOVA.

Figure 3A shows responses of SHAM and OBX rats on the active lever during the acquisition phase of WIN self-administration training. Repeated measures ANOVA revealed no significant effects over the first 8 days of training, whereas from day 9 onward a significantly higher active lever-pressing rate was observed in OBX compared with SHAM rats (repeated measures ANOVA: α < 0.001). In contrast, inactive lever-pressing rates of OBX and SHAM rats were statistically indistinguishable throughout the 30 days of training and remained constantly below 6 responses per session starting from the first week of training, with the sole exception of the initial 4 days of training (data available as Supplementary Figure 1A). This indicates that the increase in the rate of responding observed in OBX rats was not due to an unspecific effect, as further confirmed by the finding of no significant differences between OBX and SHAM animals in the basal motor activity during the self-administration daily sessions, as measured by the mean number of interruptions of the photocell beams located inside the boxes (mean activity over the maintenance phase: 989 ± 41 and 1005 ± 27 for OBX and SHAM rats, respectively).

In accordance with this, the total amount of WIN consumed by OBX rats during the maintenance phase, i.e., once animals stabilized drug intake, was significantly higher than that consumed by SHAM rats. More specifically, mean WIN intake during the last 7 days of training before extinction was significantly higher (+105%) in OBX than in SHAM rats (repeated measures ANOVA: α < 0.001) (Figure 3B). However, the percentages of rats meeting acquisition criteria for WIN self-administration in OBX and SHAM groups were similar, being 85.5 and 86.8%, respectively.

Furthermore, OBX and SHAM rats displayed clear cut differences in the time course of operant behavior even when saline was substituted for WIN, i.e., during extinction training (Figure 3C). Analysis of response on the active lever by repeated measures ANOVA showed significant differences between OBX and SHAM animals (α = 0.012 from day 1 to 7; α = 0.004 from day 8 to 14, α < 0.001 from day 15 to 28). Specifically, on the 1st day of extinction, OBX and SHAM rats reacted to saline substitution by increasing their mean active responding from 39 to 78.5 and from 16.86 to 45.17, respectively, which corresponds to +101 and +168% with respect to the last (30th) day of WIN self-administration training. Following 1-week extinction, OBX and SHAM rats reduced their active responding of −41 and −65%, respectively, with respect to day 1 extinction. Differences between OBX and SHAM groups in the responding on the active lever slightly reduced after 2 and 3 weeks of extinction training (OBX: −65 and −79%, SHAM: −78 and −90%, respectively).

**Figure 4:**
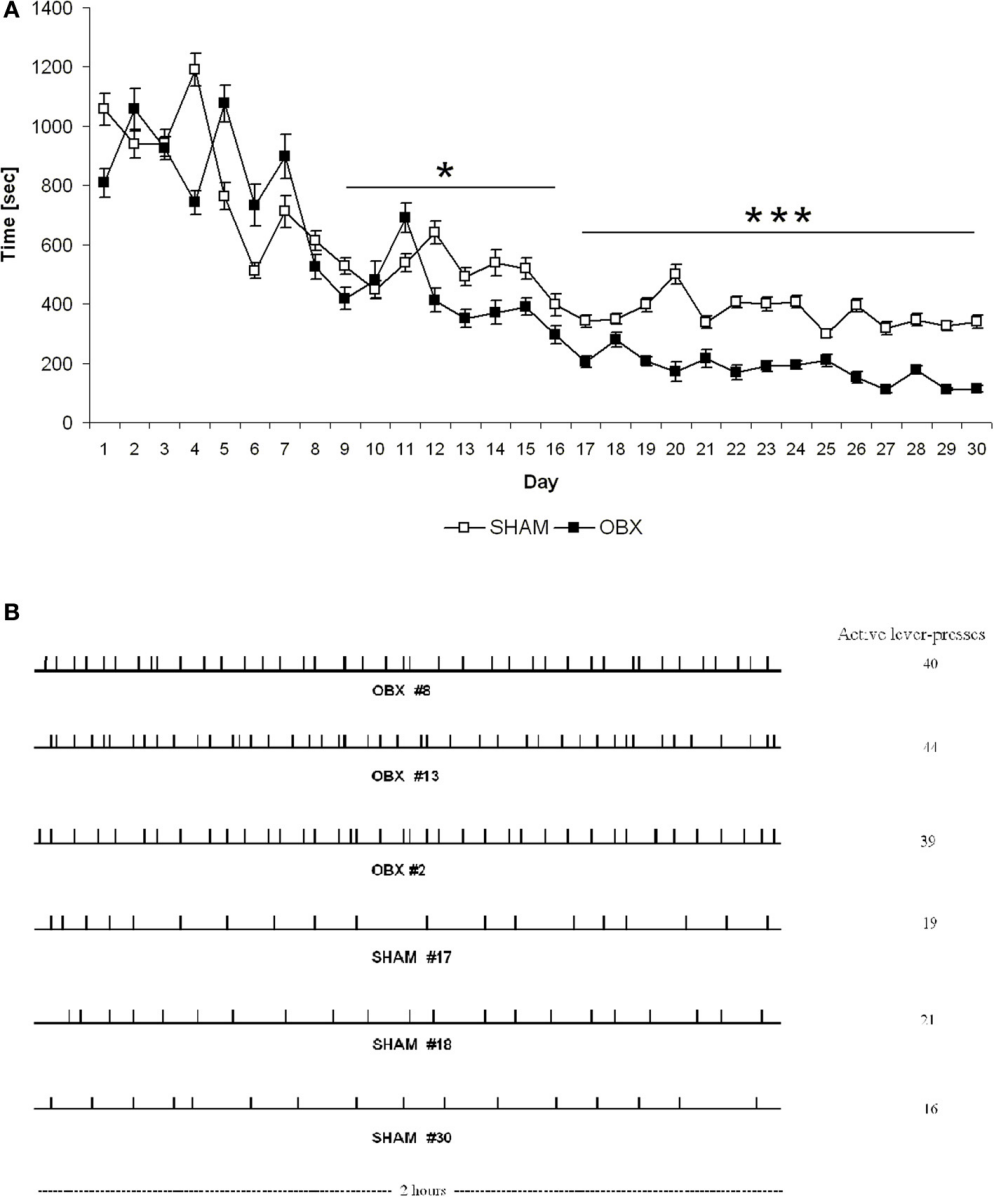
**Latency to the first active response (A) and patterns of responding (B) in SHAM and OBX Lister Hooded rats during WIN self-administration. (A)** Latency (seconds) to the first active lever presses in SHAM (*n* = 6) and OBX (*n* = 7) rats. Data are shown as means (±s.e.m.). ^*^α < 0.05 and ^***^α < 0.001, repeated measures ANOVA. **(B)** Quantitative, but not qualitative, differences in the active responding patterns between OBX and SHAM rats. Individual representative records illustrating responding patterns of OBX (first three patterns) and SHAM (last three patterns) rats on the active lever on the last day of the self-administration training (day 30). Each tick denotes the time of every response on the active lever. Cumulative numbers of active responses made over the 2 h test sessions are illustrated on the right side of each pattern.

Finally, response latency (defined as time elapsed from commencement of the experimental session until the first active lever press) was significantly different between the two groups. More specifically, response latency was shorter in OBX than SHAM rats from day 9 onward (repeated measures ANOVA: α = 0.029 from day 9 to 16; α < 0.001 from day 17 to 30) (Figure 4A), which suggests that after initial exposure to the CB_1_ receptor agonist the bulbectomized rats may be more motivated than control animals to obtain it. Moreover, analysis of temporal patterns of responses revealed quantitative but not qualitative differences between OBX and SHAM rats during self-administration training since the response rate was typically slow and evenly distributed throughout the 2 h test session in both groups (Figure 4B).

### Effect of acute pre-treatment of CGS-12066B on drug self-administration

**Figure 5:**
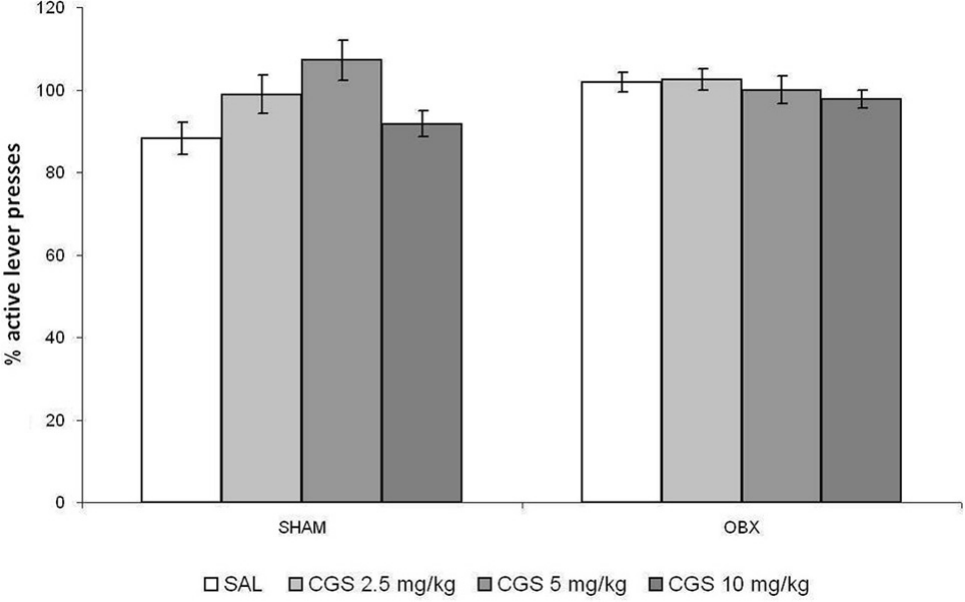
**Acute pre-treatment with CGS does not affect WIN self-administration**. Effect of acute pre-treatment with CGS-12066B on WIN self-administration in SHAM (*n* = 6) and OBX (*n* = 7) Lister Hooded rats. Data are expressed as percentage changes of active lever pressing compared to 6-day baseline (assumed as 100%). The repeated measures ANOVA did not detect a significant effect of drug treatment.

The effect of an acute administration of the serotonin 5-HT_1B_ receptor agonist CGS-12066B (CGS) was tested only after acquiring reliable WIN self-administration, i.e., once rats stabilized daily drug intake. Overall, we did not find changes in WIN self-administration after acute pre-treatment with the CGS in Lister Hooded OBX or SHAM rats. Figure 5 illustrates the percentage changes of active lever presses from the baseline after an acute challenge with CGS (2.5, 5, and 10 mg/kg) and saline control as compared to previous 6-day mean responding (i.e., baseline). The repeated measures ANOVA with OBX/SHAM group as cofactor did not detect a significant effect of drug treatment within each group nor between groups. The *post-hoc p*-values for each drug dose were as follows for SHAM: 2.5 mg/kg: *p* = 0.241; 5 mg/kg: *p* = 0.071; 10 mg/kg: *p* = 0.128, and for OBX: 2.5 mg/kg: *p* = 0.963; 5 mg/kg: *p* = 0.652; 10 mg/kg: *p* = 0.523.

**Figure 6:**
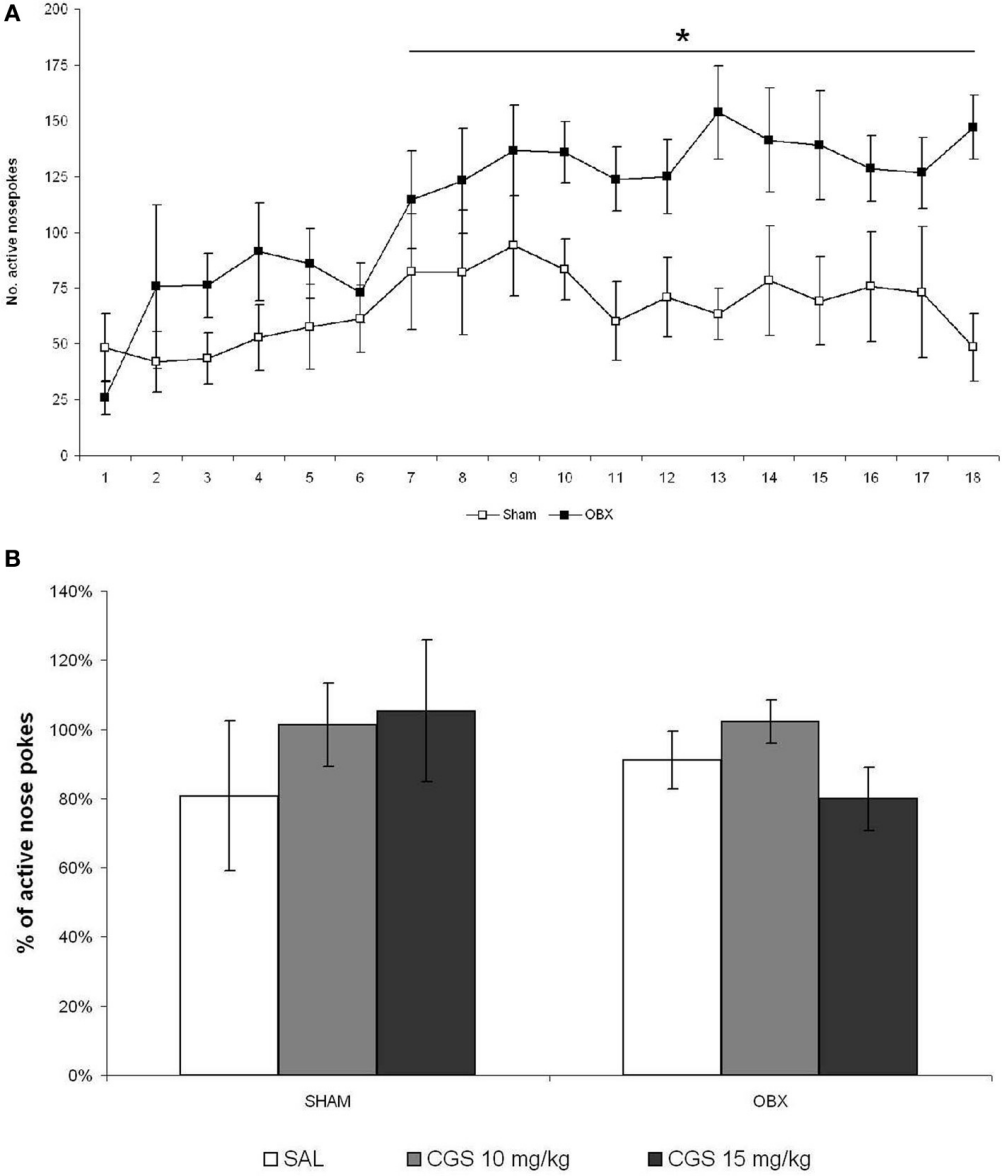
**Acute pre-treatment with CGS does not affect METH self-administration**. **(A)** METH self-administration in OBX and SHAM rats. Data expressed as daily means (±s.e.m.) of active nose-pokes in SHAM (*n* = 7) and OBX (*n* = 7) Sprague Dawley rats during methamphetamine self-administration training. Significant difference was recorded from the day 7 onwards, ^*^α < 0.05 (day 7–12: ^*^α = 0.041, day 13–18: ^*^α = 0.027, repeated measures ANOVA). **(B)** Effect of acute pre-treatment with CGS-12066B on METH self-administration. Data are expressed as percentage changes of active lever pressing compared to six-day baseline (assumed as 100%). The repeated measures ANOVA did not detect a significant effect of drug treatment.

It was reported that the effects of serotonergic drugs may differ significantly depending on the animal strain and the experimental conditions used (Horowitz et al., 1997; Uphouse et al., 2002; Miryala et al., 2013), and that not all rat strains do self-administer WIN spontaneously (Deiana et al., 2007). Moreover, the CGS compound was found to reduce amphetamine (Fletcher and Korth, 1999) and alcohol (Tomkins and O'Neill, 2000; Czachowski, 2005), but not cocaine (Parsons et al., 1996), self-administration. Thus, we decided to test the CGS compound on the self-administration of a pharmacologically different drug, such as METH, which is known to be strongly self-administered by OBX rats. We therefore tested CGS in OBX and SHAM Sprague Dawley rats self-administering METH using different response-like *operandum*, i.e., nose-poking instead of lever-pressing, and a slightly higher schedule of reinforcement (FR-2). Figure 6A illustrates responses of SHAM and OBX rats on the active hole during the acquisition and maintenance phases of METH self-administration. In line with previous findings (Kucerova et al., 2009, 2012), rats stabilized METH self-administration behavior within 14 days of training intake with a mean daily drug intake of 1.8 mg in SHAM and 3 mg in OBX animals (METH intake data shown in Supplementary Figure 2). Repeated measures ANOVA revealed no significant effects over the first 6 days of training, whereas from day 7 onward a significantly higher active nose-poking rate was observed in OBX compared with SHAM rats (repeated measures ANOVA: α < 0.05). However, while during WIN self-administration the numbers of inactive lever-presses was constantly below 5 during the maintenance phase, inactive nose-pokes during METH self-administration were higher in both OBX and SHAM rats (see Supplementary Figure 1B), an effect likely due to the activational motor effects of METH. Yet, the mean number of active nose-pokes was substantially higher than the inactive ones, which supports the specificity of animal responding for METH (preference of the active operandum during the maintenance phase was higher than 70% in all animals). Figure 6B reports the percentages of active nose pokes for METH after acute pre-treatment with saline control, 10 and 15 mg/kg of CGS-12066B compared to previous 6-day mean responding (i.e., baseline) in SHAM and OBX Sprague Dawley rats. The repeated measures ANOVA with OBX/SHAM group as cofactor did not detect a significant effect of drug treatment within each group nor between groups. The *post-hoc p*-values for each drug dose were as follows for SHAM: 10 mg/kg: *p* = 0.508; 15 mg/kg: *p* = 0.550, and for OBX: 10 mg/kg: *p* = 0.232; 15 mg/kg: *p* = 0.319. These findings indicate that, as for WIN self-administration, acute pre-treatment with the 5-HT1B receptor agonist did not modify the voluntary intake of METH.

### *In vivo* microdialysis of dopamine level in the nucleus accumbens shell of lister hooded rats

**Figure 7:**
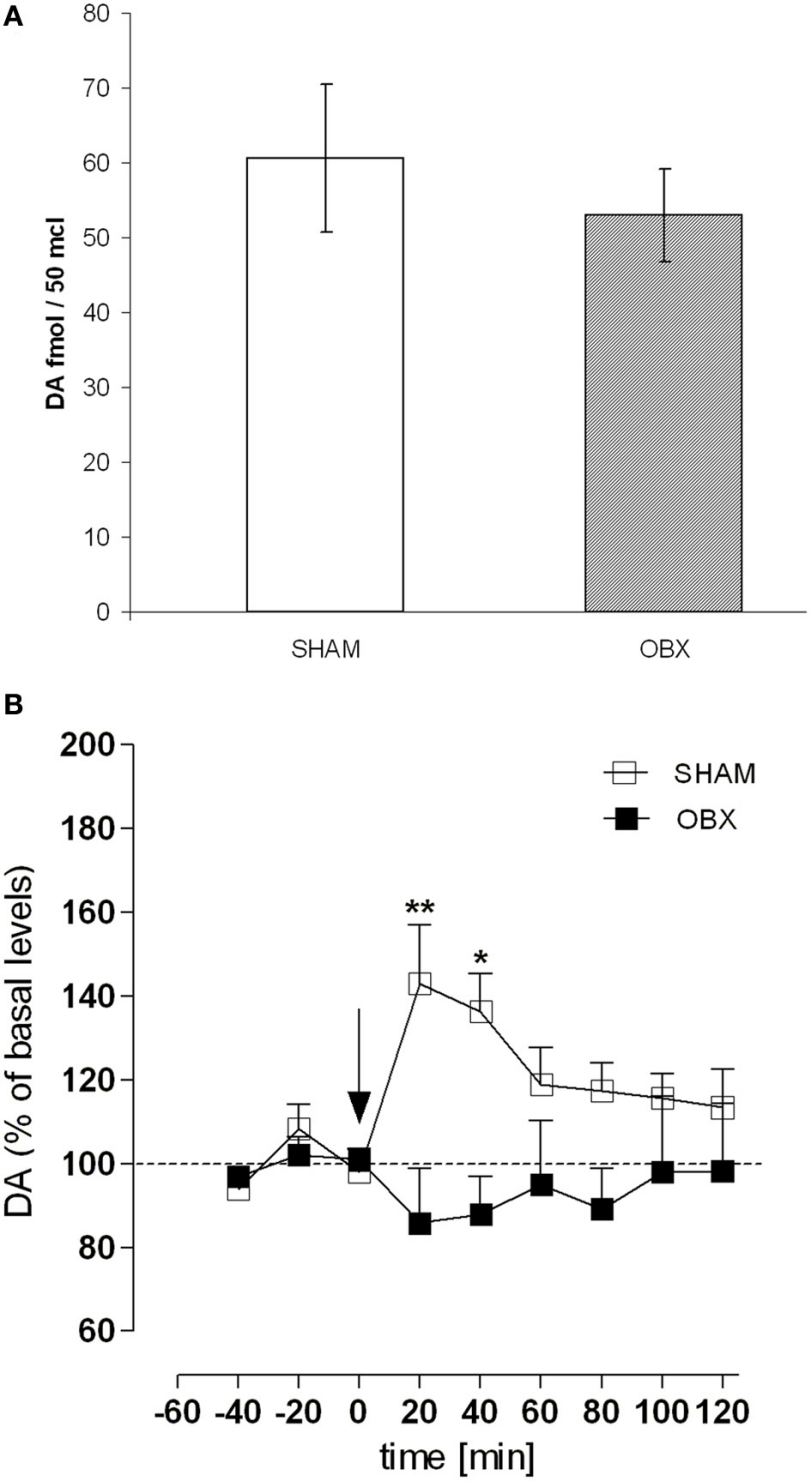
**OBX rats do not show enhanced dopamine level following acute WIN challenge**. **(A)** Basal extracellular levels (fmol/μl of dialysate, mean ±s.e.m.) of DA in the NAc shell of SHAM (*n* = 4) and OBX (*n* = 4) Lister Hooded rats. No significant difference was found between the two groups (One-Way ANOVA, *p* = 0.45). **(B)** Effect of an intravenous administration of WIN 0.3 mg/kg on DA release in the nucleus accumbens shell of SHAM (*n* = 4) and OBX (*n* = 4) Lister Hooded rats. ^*^p < 0.05 and ^*^p < 0.01, Two-Way ANOVA followed by Bonferroni's *post-hoc* test.

Figure 7 shows results from microdialysis experiment aimed at measuring the release of DA in the NAc shell of Lister Hooded SHAM and OBX rats following an intravenous injection of 0.3 mg/kg of WIN, a dose mimicking the mean amount of drug typically self-administered by naive Lister Hooded rats (Deiana et al., 2007; Fattore et al., 2007; Spano et al., 2010), and known to increase DA level in the rat NAc shell (Tanda et al., 1997). During the pre-treatment period, basal extracellular values of DA in the NAc shell did not differ significantly between the two groups (Figure 7A). As shown in Figure 7B, after WIN administration, we found a significantly increased (about +40%) extracellular DA level in SHAM rats compared to their basal level during the first 40 min after drug injection [One-Way ANOVA *F*_(8, 24)_ = 4.997, *p* = 0.0010]. However, the WIN challenge did not increase DA levels in OBX rats, in which DA levels did not significantly differ from to their previous baseline during the 2-h measurement [One-Way ANOVA *F*_(8, 24)_ = 0.3730, *p* = n.s.]. Data are expressed as mean ± s.e.m. percentage variation of basal levels. Two-Way ANOVA revealed a significant effect of treatment × time interaction [*F*_(8, 48)_ = 3.07, *p* = 0.0071; ^**^p < 0.01 and ^*^p < 0.05, Bonferroni post-test].

## Discussion

Findings of the present study demonstrated that bulbectomized rats: (i) do self-administer higher amount of the cannabinoid CB_1_ receptor agonist WIN55,212-2 than SHAM control rats, (ii) do not alter voluntary intake of the CB_1_ receptor agonist after acute pre-treatment with the serotonergic 5-HT_1B_ receptor agonist CGS12066B, and (iii) do not increase DA level in the NAc shell in response to an acute challenge with a dose of WIN (0.3 mg/kg), as SHAM rats do.

### WIN self-administration in OBX and SHAM rats

Bulbectomized rats have been previously reported to self-administer more nicotine (Vieyra-Reyes et al., 2008), amphetamine (Holmes et al., 2002), and METH (Kucerova et al., 2012) than SHAM control rats. Yet, despite clinical evidence for a significant association between smoking cannabis and major depression (Horwood et al., 2012; Lev-Ran et al., 2013), cannabimimetic drug-taking behavior was never investigated in an animal model of depression. Cannabinoid CB_1_ receptor agonists were shown to be readily self-administered by mice, rats, and monkeys (Martellotta et al., 1998; Fattore et al., 2001; Justinova et al., 2003). Notably, rate of responses was critically dependent on a variety of experimental conditions including drug unitary dose (Martellotta et al., 1998), food restriction regimen (Fattore et al., 2001), and type of *operandum* (Deiana et al., 2007). In this study, we adopted all parameters and experimental conditions that support a robust cannabinoid drug-taking behavior in the Lister Hooded rat strain (Deiana et al., 2007).

Before starting self-administration training and microdialysis experiments, we verified the development of a depressive-like phenotype in OBX lesioned animals by assessing the presence of anhedonia and hyperactive locomotor response to novel environment, which are two of the major hallmarks of this animal model of depression (Kelly et al., 1997; Song and Leonard, 2005; Romeas et al., 2009).

WIN self-administration by OBX rats significantly differed from SHAM controls as OBX animals showed higher rates of drug-associated operant responses during the maintenance phase, i.e., after initial acquisition. Indeed, although both OBX and SHAM rats needed a similar number of training sessions to acquire self-administration behavior, rates of active lever-pressing during the maintenance sessions were remarkably higher in OBX than SHAM rats. Therefore, the amount of WIN consumed by OBX rats over the 30 test sessions resulted significantly higher than in SHAM rats (mean cumulative amount of WIN over the 30-day training: 12.71 vs. 7.9 mg for OBX and SHAM, respectively). Similar rates of acquisition indicate that OBX rats required the same time of SHAM rats to stabilize their drug intake and suggest that development of a depressive-like phenotype is not associated with learning or memory deficits able to affect the acquisition of the operant task, although OBX animals have been reported to display impaired spatial learning (Song and Leonard, 2005). On the other hand, higher drug intake in OBX rats during the maintenance phase suggests that bulbectomized animals are differently responsive than SHAM controls to the cannabinoid, which might lend some support to the proposed self-medication theory of smoking cannabis to alleviate symptoms of depression (Gruber et al., 1996; Ogborne et al., 2000).

Our results are in line with the notion that OBX rats differ in the behavioral responses to acute and repeated exposure to other addictive drugs including METH (Kucerova et al., 2012), alcohol (Chiang et al., 2008), nicotine (Vieyra-Reyes et al., 2008), cocaine (Calcagnetti et al., 1996; Chambers et al., 2004), and amphetamine (Holmes et al., 2002). Notably, enhanced WIN intake by OBX rats recorded in this study was unlikely due to changes in locomotor activity since motor activity during the daily training session was not dissimilar between OBX and SHAM animals as confirmed by their similar numbers of photocell beams breaks measured within the operant boxes during daily training sessions.

Differences in the response rates on the active lever were also observed when vehicle was substituted for the CB_1_ receptor agonist. In fact, rates of responses in OBX rats were consistently higher than in SHAM rats not only when WIN was contingently available but also when it was absent, as during extinction training. A neurobiological mechanism that may contribute to the resilience of OBX rats to extinguish not-rewarded operant responses is a dysfunction of the front-cortical neuronal circuits critically involved in the inhibition of on-going activity upon withdrawal of the reinforcers (Jentsch and Taylor, 1999). This hypothesis is corroborated by the finding that OBX animals (i) are unable to adapt to environmental changes and show hyperemotional responses (Van Riezen and Leonard, 1990), (ii) exhibit impulsive-like traits (Kamei et al., 2007), and (iii) display significant increases in both CB_1_ receptor density and functionality in the prefrontal cortex (Rodriguez-Gaztelumendi et al., 2009).

### Effect of 5-HT_1B_ receptor acute stimulation on drug self-administration

In an attempt to evaluate possible mechanisms underlying the observed differences in WIN self-administration between OBX and SHAM rats, we tested the effect of a serotonin 5-HT_1B_ receptor agonist on the cannabinoid agonist intake. This choice was based on the finding that cortical and hippocampal 5-HT_1B_ receptors are critically involved in ethanol dependence and that their activation in limbic areas may attenuate amphetamine self-administration (Miszkiel et al., 2012). Moreover, a hypo-functionality of 5-HT_1B_ receptors was observed in depressed patients (Murrough et al., 2011), and a polymorphism at the 5-HT_1B_ receptor gene was found to be associated with alcoholism (Lappalainen et al., 1998). The 5-HT_1B_ receptor agonist CGS-12066B was shown to selectively decrease operant responses for ethanol (Czachowski, 2005). This compound is a full agonist with high selectivity to the 5-HT_1B_ receptor (Neale et al., 1987) and, to minor extend, to the 5-HT_1A_ receptors. The range of doses of the 5-HT_1B_ receptor agonist CGS-12066B used in this study was shown to be effective in acute in altering aggressive (De Boer and Koolhaas, 2005) and sexual behavior (Maciag et al., 2006) as well as some reward-related behaviors, such as DA-mediated reinforcement (Parsons et al., 1996). However, it did not affect cocaine self-administration in rats (Parsons et al., 1996), similarly to our findings on WIN self-administration.

Importantly, both the effects of serotonergic drugs (Horowitz et al., 1997; Uphouse et al., 2002; Miryala et al., 2013) and WIN self-administration behavior (Deiana et al., 2007) have been reported to greatly vary depending on rat strain and/or experimental parameters and procedures adopted. Moreover, the CGS compound was found to affect drug self-administration selectively, as it decreases alcohol (Grant et al., 1997; Maurel et al., 1999; Tomkins and O'Neill, 2000; Czachowski, 2005) and d-amphetamine (Fletcher and Korth, 1999), but not cocaine (Parsons et al., 1996) intake. Thus, we decided to test CGS-12066B on the self-administration of METH for which OBX rats are known to display higher responding level than SHAM rats (Kucerova et al., 2012), as for WIN. Yet, acute pre-treatment with the 5-HT_1B_ receptor agonist CGS-12066B did not significantly alter the intake of METH neither in OBX and SHAM Sprague Dawley rats nor in intact Wistar rats as recorded in our earlier unpublished pilot experiment (data available as Supplementary Figure 3).

Thus, the present findings indicate that WIN and METH, like cocaine (Parsons et al., 1996) self-administration, are not affected by acute stimulation of the 5-HT_1B_ receptor. Serotonin 5-HT_1B_ receptors are expressed throughout the brain of rodents. They are located in the axon terminals of both 5-HTergic and non-5-HTergic neurons where they act as inhibitory autoreceptors or heteroreceptors, respectively, (Barnes and Sharp, 1999; Moret and Briley, 2000; Pytliak et al., 2011; Cai et al., 2013), and have been difficult to study because of the diversity of their localization and the absence of highly selective receptor antagonists. Findings from the present study do not allow excluding the possibility that a chronic rather than an acute stimulation of 5-HT_1B_ receptors might alter WIN and METH self-administration. Thus, future studies will evaluate the effects of chronic stimulation of 5-HT_1B_ receptor by CGS-12066B, administered systemically or locally, on WIN and METH self-administrations.

### Reduced sensitivity of OBX rats to the WIN stimulation effect on dopamine release in the nucleus accumbens shell

Enhanced drug self-administration can be linked to a dysfunction in the reward system, which is very likely to occur in OBX animals given the chemical and molecular changes that bulbectomy induces in several neurotransmitter systems, including the dopaminergic one (Masini et al., 2004; Sato et al., 2010), a major component of the brain reward system.

In intact animals, acute administration of WIN55,212-2 is known to increase extracellular DA levels in the NAc of freely moving rats (Gardner and Lowinson, 1991; Cheer et al., 2004; Polissidis et al., 2013). Moreover, DA content in the rat NAc shell was shown to increase appreciably in respect to basal values during WIN self-administration (Fadda et al., 2006). According to this, the SHAM rats in this study significantly increased accumbal DA levels in response to an intravenous administration of a dose of WIN, 0.3 mg/kg, very similar to that daily self-administered by rats. Notably, the same dose of WIN enhances DA levels in the NAc shell of drug-naïve Sprague Dawley rats (Tanda et al., 1997). However, in our experiment WIN did not enhance DA levels in bulbectomized rats.

To explain this finding it could be of help to consider the multiple dysregulations that OBX induce in the endocannabinoid brain system. Cannabinoid CB_1_ receptor density is significantly increased in OBX rats in the medial prefrontal cortex (mPFC) and amygdala while it does not change in the caudate-putamen, hippocampus, and dorsal raphe nucleus (Rodriguez-Gaztelumendi et al., 2009). CB_1_ receptor function is significantly enhanced in the mPFC of OBX animals with respect to SHAM controls, but not in other brain regions with the exception of a slight, not significant, increase in the amygdala (Rodriguez-Gaztelumendi et al., 2009). Moreover, OBX does not affect DA D1- and D2-like receptors in the NAc (Sato et al., 2010). Thus, potential changes in the number or function of either the CB_1_ or the dopaminergic receptors following OBX are unlikely to account for the absence of WIN-induced effect on DA release in the NAc of OBX rats. On the other hand, there is no clear evidence that DA release in the reward pathway depends on DA receptors. Instead, it is known that midbrain DA neurons produce endocannabinoids which retrogradely influence the glutamate and GABA projections and thus regulate the inhibitory and excitatory inputs to the reward circuit (Melis and Pistis, 2007). Glutamatergic and GABAergic systems are both dysregulated in the OBX model leading, among others, to a hyperactive response to novel environment (Song and Leonard, 2005). These dysregulations may contribute to the differential reactivity of the mesolimbic dopaminergic system in OBX rats. Thus, future studies will be performed to assess whether chronic treatment with this dose of WIN (0.3 mg/kg) as well as acute challenges with higher WIN doses may elicit an increase in DA levels in the NAc shell of OBX rats, and to evaluate CB_1_ and DA receptor densities.

To summarize, this study demonstrated that OBX rats self-administer more cannabinoid agonist than SHAM control rats, and that WIN taking behavior is not significantly affected by acute stimulation of 5-HT_1B_ receptors. The close anatomical and functional association between the olfactory bulbs and the limbic system may help to understand why OBX rats differ from SHAM rats in drug self-administration behavior. The neurons of the olfactory bulbs are widely interconnected with other brain regions including cortical areas and limbic nuclei (Song and Leonard, 2005). The projections to these nuclei may be particularly relevant to changes in emotional and reward-related behavior in bulbectomized rats. Removal of the olfactory bulbs may alter, if not disrupt, the activity in brain circuits, particularly those influencing the dopaminergic system which is critical for processing drug taking and seeking behaviors. As OBX rats, contrary to SHAM, did not display a significant increase of DA levels in the NAc shell after an acute WIN challenge, we hypothesize that a depressive-like state may alter the rewarding effects of the drugs.

In conclusion, our findings showed that OBX markedly affects self-administration of cannabinoid CB_1_ receptor agonist, possibly through a reduction of its rewarding effects to which animals compensate by increasing WIN intake. A decreased DA neurotransmission in the NAc shell might contribute to this compensatory behavior. Thus, a follow-up study will evaluate (i) a dose-response effect of acute and chronic WIN and METH administration on NAc shell DA levels in OBX and SHAM rats, and (ii) DA levels after immediate (24 h), short-term (1 and 2 weeks), and long-term (4-weeks) cessation from chronic drug exposure. Future studies will also evaluate whether OBX and SHAM rats also differ in the reinstatement of cannabinoid-seeking behavior trigger by drug, cue, or stress primings.

## Author contributions

Petra Amchova was responsible for the induction of OBX model in the Czech Republic and its transfer to the Italian laboratory; she collected the data in the Czech Republic and processed them for analysis, and wrote a substantial part of the introduction and methods sections of the manuscript. Jana Kucerova developed the original idea and organized the experimental work in the Czech Republic, and wrote a substantial part of the introduction, methods, results and discussion sections of the manuscript. Valentina Giugliano was responsible for the behavioral testing and performance of the OBX surgery in Italy and collection of data, and she cross-checked the materials and methods section of the manuscript. Zuzana Babinska was responsible for the behavioral testing in the Czech Republic and contributed to microdialysis experiments in Italy, and she cross-checked the materials and methods section of the manuscript. Mary Tresa Zanda contributed substantially to behavioral testing and OBX surgery in Italy and collection of data, and she cross-checked the materials and methods section of the manuscript. Maria Scherma was responsible for the microdialysis experiment in Italy and for the related statistical data analysis and graphical presentation of data. Ladislav Dusek performed the statistical data analysis and contributed substantially to the results section and graphical presentation of the results. Paola Fadda organized and supervised the microdialysis experiment in Italy and was involved in the analysis and discussion of the data, and contributed to the final version of the manuscript. Vincenzo Micale established the collaboration of the two departments, was involved in discussion of the data and contributed to the final version of the manuscript. Alexandra Sulcova was involved in the design of the study and discussion of the data, and contributed to the final version of the manuscript. Walter Fratta was involved in the analysis and discussion of the data, and contributed to the final version of the manuscript. Liana Fattore organized and supervised the experimental work in Italy and enabled the transfer of the OBX technique; she wrote a substantial part of the introduction, methods, results and discussion sections of the manuscript.

### Conflict of interest statement

The authors declare that the research was conducted in the absence of any commercial or financial relationships that could be construed as a potential conflict of interest.
