# Supplementary material for: Enhanced self-administration of the CB1 receptor agonist WIN55,212-2 in olfactory bulbectomized rats: evaluation of possible serotonergic and dopaminergic underlying mechanisms
Source: Front Pharmacol. 2014 Mar 20;5:44. doi: 10.3389/fphar.2014.00044 (PMC3960502; doi:10.3389/fphar.2014.00044)

SUPPLEMENTARY MATERIAL FOR PAPER:

Enhanced self-administration of the CB1 receptor agonist WIN55,212-2 in olfactory bulbectomized rats: evaluation of possible serotonergic and dopaminergic underlying mechanisms

Authors: Petra Amchova, Jana Kucerova, Valentina Giugliano, Zuzana Babinska, Mary Tresa Zanda, Maria Scherma, Ladislav Dusek, Paola Fadda, Vincenzo Micale, Alexandra Sulcova, Walter Fratta, Liana Fattore

Frontiers in Pharmacology

Category: Original research

Research Topic: Addictive drugs targeting GPCRs: new cross-talk mechanisms

SUPPLEMENTARY FIGURE LEGENDS:

Supplementary Figure 1. OBX and SHAM rats do not differ in inactive *operandum* responding. A) Mean number of inactive lever presses over the acquisition and maintenance period (30 days) in SHAM (n=7) and OBX (n=7) Lister Hooded rats during WIN self-administration. Data are shown as means (±SEM). Not significant differences, repeated measures ANOVA, $\alpha = 0.768$. **B)** Mean number of inactive nosepokes over the acquisition and maintenance period (18 days) in SHAM (n=7) and OBX (n=7) Sprague Dawley rats during METH self-administration. Data are shown as means (±SEM). The repeated measures ANOVA did not detect a significant effect of drug treatment.

Supplementary Figure 2. OBX rats display enhanced METH self-administration behaviour. Mean number of infusions in SHAM (n=6) and OBX (n=7) Sprague Dawley rats during METH self-administration. Each infusion contains 0.08 mg/kg METH. The mean number of infusions during the whole self-administration training was 23 in SHAM and 35.8 in OBX (approx. 1.8 and 3 mg/kg METH respectively). Data are shown as daily means (±SEM) and start to differ significantly from day 7 onwards, * $\alpha < 0.05$, repeated measures ANOVA.

Supplementary Figure 3. Acute pre-treatment with CGS does not affect METH self-administration in intact Wistar rats. Effect of acute pre-treatment with CGS-12066B on methamphetamine self-administration in intact Wistar rats (n=5). Data are expressed as percent of active lever pressing compared to six-day baseline (assumed as 100%). The repeated measures ANOVA did not detect a significant effect of drug treatment.

Supplementary Figure 1: OBX and SHAM rats do not differ in inactive operandum responding.

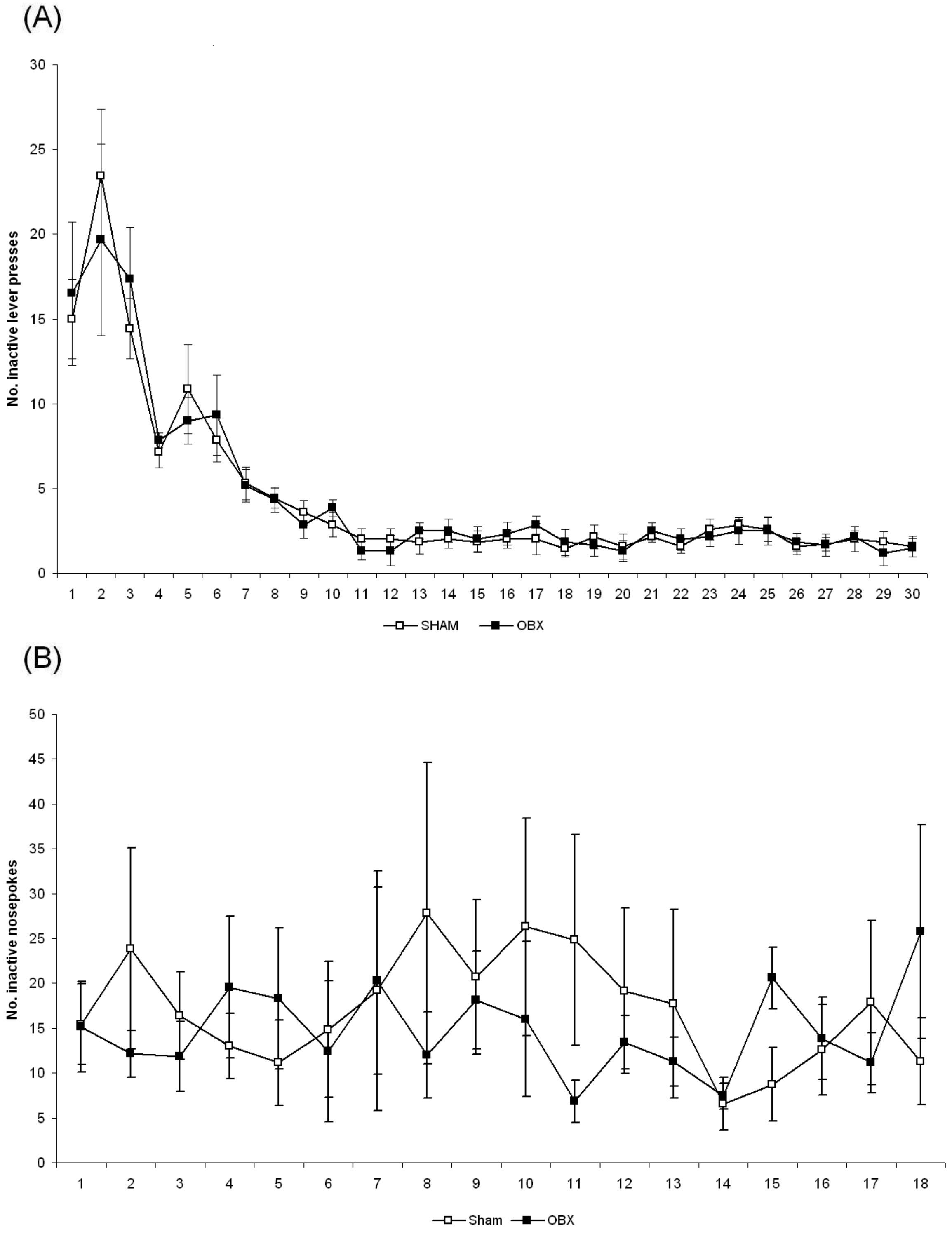

Supplementary Figure 2: OBX rats display enhanced METH self-administration behaviour.

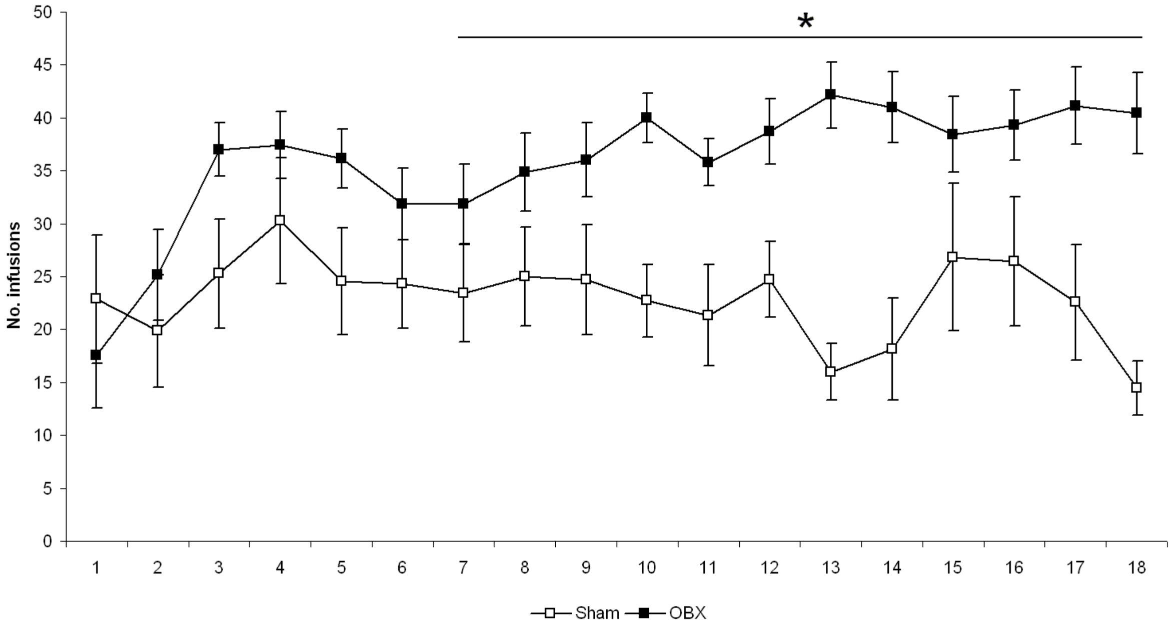

Supplementary Figure 3: Acute pre-treatment with CGS does not affect METH self-administration in intact Wistar rats.

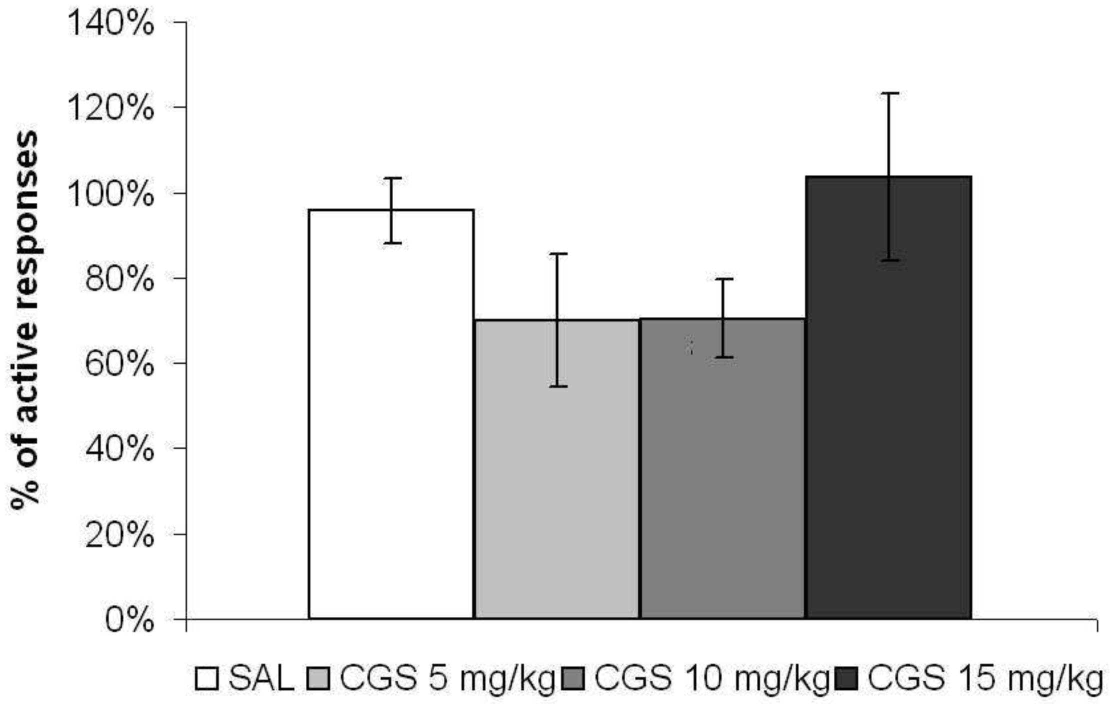

Supplement: Supplementary file 1 [file Presentation1.PDF]
